# Treatment of Capsular Contracture in Previously Irradiated Breasts Implants and Expanders with the Use of Porcine Acellular Dermal Matrices: Outcomes and Complications

**DOI:** 10.3390/jcm13185653

**Published:** 2024-09-23

**Authors:** Andrea Vittorio Emanuele Lisa, Riccardo Carbonaro, Manuela Bottoni, Giulia Colombo, Marika Gentilucci, Valeriano Vinci, Edvin Ostapenko, Luca Nicosia, Francesca De Lorenzi, Mario Rietjens

**Affiliations:** 1 Department of Plastic and Reconstructive Surgery, European Institute of Oncology, IRCCS, Via Ripamonti, 435, 20141 Milan, Italy; carbonaro.doc@gmail.com (R.C.);; 2 Department of Medical Biotechnology and Translational Medicine (BIOMETRA), Reconstructive and Aesthetic Plastic Surgery School, University of Milan, Via Festa del Perdono 7, 20122 Milan, Italy; 3 PhD Program in Applied Medical-Surgical Sciences, Department of Surgical Sciences, University of Rome “Tor Vergata”, Cracovia n. 50, 00133 Rome, Italy; 4 Plastic and Reconstructive Surgery Department, University of Milan, Via Festa del Perdono 7, 20122 Milan, Italy; 5 Department of Biomedical Sciences, Humanitas University, 20072 Milan, Italy; 6 Faculty of Medicine, Vilnius University, M. K. Čiurlionio g. 21, 03101 Vilnius, Lithuania; 7 Breast Imaging Division, IEO European Institute of Oncology IRCCS, Via Ripamonti, 20141 Milan, Italy; luca.nicosia@ieo.it

**Keywords:** breast reconstruction, radiotherapy, acellular dermal matrix (ADM)

## Abstract

**Background**: Radiation therapy is a crucial component of breast cancer treatment. However, it is well known to increase the risk of unsatisfactory cosmetic outcomes and higher complication rates. The aim of this study is to provide further insight into the use of acellular dermal matrices (ADMs) for the prevention of capsular contracture. **Materials and Methods**: This single-center, retrospective study analyzed irradiated patients who underwent post-mastectomy, ADM-assisted implant reconstructions. Of the 60 patients included, 26 underwent expander-to-implant substitution after radiotherapy (Group A), while 34 required implant replacement due to capsular contracture following radiotherapy (Group B). The primary objective was to evaluate the effectiveness of ADMs in reducing reconstructive failures, complications, and capsular contracture after breast irradiation. **Results**: We recorded a total of 15 complications and four implant losses. Reconstructive failures were attributed to implant exposure in two cases, full-thickness skin necrosis in one case, and severe Baker grade IV contracture in one case. Both Group A and Group B showed a significant decrease in postoperative Baker grades. US follow-up was used to demonstrate ADM integration with host tissues over time. **Conclusions**: Based on our findings, the use of ADM in selected cases appears to be a viable option for treating and preventing capsular contracture in irradiated breasts. This approach is associated with relatively low complication rates, a low rate of reconstructive failure, and satisfactory cosmetic outcomes and can be applied both in breast reconstructed with implants and with expanders.

## 1. Introduction

Breast cancer is the most common cancer in women, with over 2 million new cases diagnosed in 2020 [1]. Following oncologic surgery, an increasing number of women opt for breast reconstruction [2]. Radiation therapy is a fundamental component of the multidisciplinary treatment of breast cancer [3]. However, it is well established that, particularly in implant-based reconstructions, radiation therapy is associated with poorer cosmetic outcomes and a significant increase in complications, especially capsular contracture [4], as well as a higher rate of reconstructive failure [5].

Several strategies have been explored to minimize complications in irradiated breasts. As a result, autologous reconstruction has proven to be the most effective approach and is generally considered the preferred method in such cases. Nevertheless, implant-based breast reconstruction is still performed in selected cases, and acellular dermal matrices (ADMs) have been used to enhance outcomes [6,7,8,9,10]. It has been suggested that ADMs may have a protective effect, reducing complications and lowering the rate of capsular contracture. Consequently, ADMs may serve as a valuable tool in high-risk scenarios, such as irradiated breasts [11,12,13,14].

The claim by some authors regarding the potential of ADMs to reduce capsular contracture has been widely documented in the literature, showing promising results in decreasing or preventing recurrence of this complication [14]. Recent research is also shedding light on the immunological and biological mechanisms underlying the effectiveness of ADMs in this context [15].

In this study, we retrospectively analyzed our single-center experience with the use of acellular dermal matrices (ADMs) in previously irradiated breasts. Current literature is notably lacking in evidence regarding alternatives to autologous reconstruction in this context. Therefore, the primary objective of this study was to evaluate the feasibility of ADM-assisted breast reconstruction in irradiated breasts as a potential alternative to autologous reconstruction, focusing on the rates of reconstructive failure, complications, and recurrence of capsular contracture. Additionally, we assessed the aesthetic outcomes of ADM-based reconstruction and compared the results between direct-to-implant reconstructions and two-stage reconstructions involving expanders and implants.

## 2. Materials and Methods

### 2.1. Patients and Study Design

From our hospital registry, we retrospectively identified all patients who underwent implant-based reconstruction using Native^®^ acellular dermal matrix ADM after radiotherapy between June 2018 and April 2022. The study was conducted in accordance with the Declaration of Helsinki, and all patients provided written informed consent prior to enrollment.

Our cohort included only women who had previously undergone mastectomy followed by immediate reconstruction with expanders or definitive implants, followed by adjuvant radiotherapy. Therefore, the series comprised patients requiring either expander-to-implant substitution (Group A), or revision surgery for capsular contracture (Group B). In all cases, Native^®^ ADM was used during the second surgery.

Native^®^ ADM is a 0.6 mm-thick collagen matrix with a rectangular shape and a continuous surface. Derived from pig dermis, it undergoes a deantigenation process to remove all animal cells, leaving only the extracellular matrix, ensuring biocompatibility and eliminating the risk of rejection when implanted. The ADM is non-crosslinked, provided without preservatives, freeze-dried for optimal preservation, and sterilized using ethylene oxide. Prior to implantation, it must be rehydrated in a saline solution at room temperature for at least 5 min.

Patients considered candidates for autologous reconstruction after irradiation (such as those with severe radiation damage, excessively thin skin, or implant exposure after radiotherapy) were excluded. At our center, autologous or combined autologous-implant reconstruction is the standard of care in cases of irradiation. However, in selected cases where skin flaps appear thick and well-vascularized, ADM was proposed following capsulotomy and anterior capsulectomy. Patients who did not receive Native^®^ ADM, as well as those who underwent Native^®^-assisted implant reconstruction after conservative treatment or delayed reconstruction following mastectomy, were excluded.

A dedicated database was established, including 60 patients (with 60 breasts reconstructed using ADM). We collected patient demographics such as age, body mass index (BMI), smoking status, and comorbidities. Additionally, surgical details, including the type of mastectomy and reconstruction, were recorded. The database also included information on tumor histology, treatment modalities (including chemotherapy and radiotherapy), and preoperative capsular contracture grade. Clinical outcomes, such as postoperative complications, the rate of reconstructive failure, and the incidence and severity of capsular contracture recurrence, were also documented.

Patients in the series were divided into two subgroups: Group A consisted of patients who had undergone mastectomy with two-stage reconstruction, where a tissue expander was placed in a total submuscular pocket and radiotherapy was administered after expansion completion. These patients were included in the study if they developed severe capsular contracture with the expander in place. They underwent a second-stage reconstruction involving the replacement of the tissue expander (TE) with a permanent implant at least three months after completing radiotherapy (RT), during which ADM was placed to minimize the development of capsular contracture. Group B consisted of patients who developed capsular contracture following mastectomy and direct-to-implant reconstruction, with the implant placed in either a total or partial submuscular pocket (i.e., reconstruction with a definitive implant during the same surgical procedure as the mastectomy). Adjuvant post-mastectomy radiotherapy (PMRT) was performed after reconstruction. All patients in Group B had developed grade 3 or 4 capsular contracture and were treated with capsulotomy and/or capsulectomy, implant replacement, and ADM placement to reduce the risk of capsular contracture recurrence.

Postoperative complications were classified as major or minor. Major complications included those requiring implant removal, such as full-thickness skin flap necrosis, implant exposure, and Baker IV capsular contracture. Minor complications included superficial skin flap necrosis, hematoma/seroma not requiring surgical revision, post-mastectomy pain syndrome (PMPS), and implant malposition. Only patients with a minimum follow-up of one year were included in the study.

### 2.2. Surgical Technique and Perioperative Care

All patients were treated by experienced surgeons under the supervision of the senior surgeon, with all procedures performed under general anesthesia.

In both groups, the previous mastectomy scar was used to access the implant pocket. After removing the implant (either expander or permanent), a circumferential capsulotomy and anterior capsulectomy were performed, as this is the standard practice at our center when severe capsular contracture is present. This differs from the capsulotomy and inferior pole capsulectomy performed when no capsular contracture is observed during implant exchange. Throughout the procedure, the surgeon carefully assessed and preserved the vascularization of the mastectomy flaps.

Meticulous hemostasis was achieved, and a silicone drain (Blake Silicone Drains, Ethicon, INC, Somerville, NJ, USA) was placed. The implant pocket was irrigated with saline solution prior to implant placement; no antibiotics or antimicrobials were used for irrigation. This is due to strict regulations from the antibiotic resistance control committee at our institution, which advises against the routine use of antibiotics for implant pocket irrigation.

The approximate implant size was preselected based on the patient’s breast width and preferences, but the final implant size was determined intraoperatively using a sizer to achieve maximum symmetry.

Next, the Native^®^ ADM was positioned between the skin envelope and the implant at the inferior pole of the breast. The ADM inset technique is straightforward: the ADM is tailored with scissors to fit the shape and size of the lower half of the chosen implant. It is then placed over the lower portion of the implant, so that it lies between the implant and the pectoral muscle in the inferomedial quadrant, and between the implant and mastectomy flap in the inferolateral quadrant. The ADM is not sutured to the skin or muscle. In our experience, careful handling and placement of suction drains is sufficient to allow the ADM to adhere to the surrounding tissue without displacement.

Preoperative antibiotic prophylaxis with 2 g of cefazolin was administered, and patients continued broad-spectrum antibiotic therapy at home for one week. The drain was removed after two consecutive days with less than 30 cc/day of serous output.

US examination:

Follow-up visits were scheduled at 1 week, 2 weeks, 3 months, 6 months, and 1 year postoperatively. In the early postoperative period, US was used to rule out seromas, which are known to be associated with the use of ADM. Subsequent yearly consultations and ultrasound checks were arranged. These US were performed in the context of the standard post-breast reconstruction follow-up protocol, however, the US was also used to assess ADM integration with host tissue.

US was performed bilaterally using a Canon Diagnostic Ultrasound System—Aplio i800 scanner with 5–18 MHz linear probes and presets dedicated to breast parenchyma evaluation. A specific focus was on the implant evaluation for any periprosthetic fluid collection or rupture signs and ADM.

Image Interpretation and Data Analysis

US examinations were evaluated by dedicated radiologists with more than five years of experience in breast imaging. At the time of evaluation, they were aware of the patient’s surgical treatment, clinical history, or subsequent post-surgical complications. ADM was detected as peri-capsular hypoechoic thickening (up to approximately 2 mm) at early postop follow-up.

At the one-year follow-up, peri-capsular hypoechoic thickening persisted in 50% of patients (30/60) at the US; the matrix was no longer recognisable in 30% of patients (18/60), and pseudonodular peri-capsular images were more prominently evident in 20% of patients (12/60).

This follow-up protocol was adhered to for all patients, except in cases of complications, which sometimes required additional visits. All patients included in the study had a minimum follow-up period of 1 year.

### 2.3. Aesthetic Results and Surgeon Evaluation

Two surgeons independently and blindly evaluated the quality of breast reconstructions based on several factors, including breast shape and volume, the position of the inframammary fold, scar quality, symmetry with the contralateral breast, and overall appearance. The evaluation also considered tissue quality, the degree of capsular contracture, and the extent of skin damage post-irradiation.

The overall aesthetic outcomes were categorized into four groups based on these parameters: good, medium, poor, and not assessable (in cases where the implant was removed due to failure).

### 2.4. Statistical Analysis

Data analysis was performed using IBM SPSS Version 24 (IBM Corp., Armonk, NY, USA). Associations between categorical variables and complication rates were assessed using the χ^2^ test, or Fisher’s exact test. Mean values and standard deviations were calculated for both groups, and differences between group means were tested using analysis of variance (ANOVA), and *p*-values were calculated for each outcome, with a significance threshold of *p* < 0.05 considered statistically significant

## 3. Results

Between June 2018 and April 2021, 60 patients met the inclusion and exclusion criteria and were included in the study. The cohort consisted of 26 patients undergoing second-stage expander-to-definitive implant reconstruction (Group A, 26 breasts), and 34 patients requiring capsular revision and implant replacement (Group B, 34 breasts). Native^®^ ADM was used in both groups.

The mean age at surgery was similar between the two groups, with 52.6 years in Group A and 51.8 years in Group B. The mean BMI was 25.5 in Group A and 24.4 in Group B. None of the patients had diabetes, autoimmune diseases, or were current users of corticosteroids. A difference in smoking habits was noted despite not being statistically significant: a total of 11% of patients in Group A were smokers, compared to 0% in Group B. Nipple- and skin-sparing mastectomies were evenly distributed between the two groups, as were the types of axillary surgery and chemotherapy. Most patients received anatomical textured implants, with a mean size of 445 cc in Group A and 448 cc in Group B. Detailed characteristics of the study population are provided in Table 1 and Table 2.

### 3.1. Major Complications—Reconstructive Failure

In total, we recorded 15 complications (25%) in our series (Table 3). Of the 60 reconstructed breasts, four experienced implant loss, corresponding to a rate of 6.6% (Table 3). Reconstructive failure occurred exclusively in Group A (irradiated expander group), with a failure rate of 15.3% (four out of 26 patients), whereas no failures were observed in Group B (irradiated implant group).

The primary cause of failure was implant exposure, affecting two out of 26 patients. Additionally, full-thickness mastectomy flap necrosis was noted in one case, and grade IV capsular contracture was observed in another. Among the patients who experienced implant failure, two (50%) underwent autologous reconstruction (latissimus dorsi LD, Transverse rectus abdomis myocutaneus flap TRAM, or deep inferior epigastric perforator flap DIEP flap), while two patients declined further surgical interventions.

### 3.2. Minor Complications

Minor complications were recorded in 11 cases (18.3%): seroma in three cases (5%), superficial skin necrosis in three cases (5%), post-mastectomy pain syndrome (PMPS) in two cases (3.2%), hematoma in one case (1.6%), and implant rotation in one case (1.6%). Importantly, no infections were observed in our case series.

In detail, Group A reported four minor complications: two cases of superficial skin necrosis (one occurring in a smoker), one case of seroma, and one case of hematoma. In Group B, we observed two cases of superficial skin necrosis, two cases of seroma, two cases of PMPS, and one case of implant rotation.

### 3.3. Capsular Contracture

Prior to reconstruction with ADM, the degree of capsular contracture was assessed in the 34 patients who had an implant in place (Group B) (Table 3). In Group A, the preoperative Baker grade could not be determined due to the temporary presence of a tissue expander at the time of surgery. Postoperatively, the Baker grades in Group A were as follows: grade I in 4/26 (15.4%), grade II in 17/26 (65.4%), grade III in 2/26 (7.7%), and grade IV in only 1/26 (3.8%) cases. Capsular contracture could not be assessed in the two cases of implant loss.

We observed a significant reduction in the degree of postoperative capsular contracture, with a very low recurrence rate. In Group B, the pre-reconstruction Baker grades were III in 26/34 patients (76.5%) and IV in 8/34 (23.5%). After implant replacement, the Baker grades were: I in 2/34 (5.9%), II in 29/34 (85.3%), and III in 1/34 (2.9%). Most importantly, there were no cases of postoperative grade IV contracture, and only one case of grade III contracture was recorded. In the two cases of implant loss, postoperative capsular contracture could not be evaluated.

A slightly higher rate of capsular contracture was observed in Group A (irradiated expander group) compared to Group B (irradiated implant group).

### 3.4. Aesthetic Results

**Figure 1:**
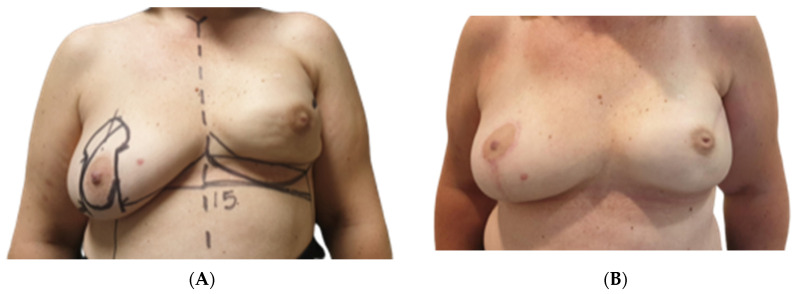
(**A**) Preoperative photograph of a 56-year-old patient who declined autologous breast reconstruction and was included in group B. History of right skin reducing mastectomy and DTI reconstruction, followed by radiotherapy, then affected by capsular contraction and implant dislocation. Patient underwent capsulotomy and anterior capsulectomy, implant exchange and ADM positioning. (**B**) Postoperative photograph at one-year follow-up of a 56-year-old patient who declined autologous breast reconstruction and was included in group B.

**Figure 2:**
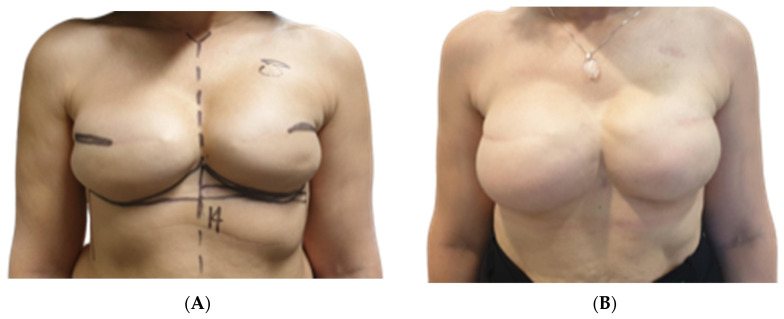
(**A**) Preoperative photograph of a 50-year-old patient included in group A. History of left nipple sparing mastectomy and tissue expander reconstruction followed by adjuvant radiotherapy. During expander-to-implant substitution after anterior capsulectomy and capsulotomy we applied ADM in order to increase soft tissue coverage and prevent capsular contraction development. (**B**) Postoperative photograph of 50-year-old patient included in group A at one-year follow-up.

**Figure 3:**
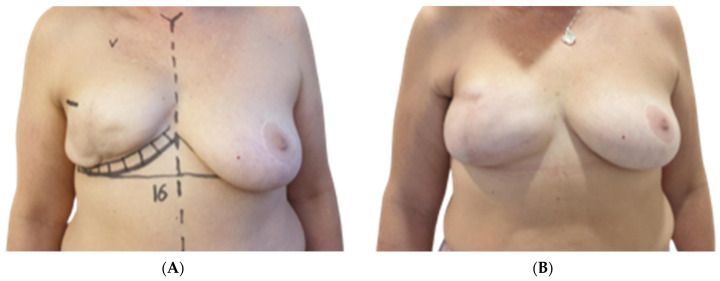
(**A**) Preoperative photograph of 49-year-old patient included in group B. History of bilateral DTI breast reconstruction followed by left breast adjuvant radiotherapy. Patient referred to our hospital for left Baker grade IV capsular contraction with functional symptoms. Underwent anterior capsulectomy, bilateral implant exchange and left ADM positioning to reduce capsular contraction and related symptoms. (**B**) Postoperative photograph of 49-year-old patient included in group B at one-year follow-up.

Both surgeons evaluating the outcomes reported a high level of satisfaction with Native^®^ ADM-assisted reconstructions, as shown in Table 4. Specifically, they were highly satisfied with the aesthetic results in 45 out of 60 cases (75%), moderately satisfied in eight cases (13.3%), and unsatisfied in two cases (3.3%). In four cases (6.7%), the aesthetic result could not be assessed due to reconstructive failure. The least satisfactory aesthetic outcomes were observed in Group A, where two out of 26 breasts (7.7%) were rated as poor. Additionally, four out of 26 cases (15.4%) were excluded from the formal aesthetic evaluation due to reconstructive failure; however, the surgeons informally rated these as “very poor” (Figure 1A,B, Figure 2A,B and Figure 3A,B).

In this comparative analysis, Group B demonstrated significantly better outcomes than Group A. Data were analyzed using the weighted kappa (wk) statistic, and interrater reliability was high, with values exceeding 0.9.

## 4. Discussion

The authors acknowledge that radiotherapy significantly increases the complication rate in implant-based breast reconstructions, ultimately leading to poorer aesthetic outcomes [16]. Chetta et al. further support this claim, reporting a 29.4% failure rate in irradiated breasts following implant reconstruction, compared to 4.3% after autologous reconstruction [17]. However, some patients opt for implant-based or hybrid reconstructions with irradiation due to personal preference, limited availability of autologous tissue, or lack of access to surgeons skilled in microsurgery. Additionally, the need for postoperative irradiation is not always predictable at the time of immediate reconstruction.

Autologous fat grafting combined with alloplastic reconstruction (referred to as “hybrid reconstruction”) has been described in the literature as improving outcomes in patients undergoing radiotherapy [18,19]. Further scientific research is needed to reduce the risk of complications in patients receiving radiotherapy who also seek implant-based reconstruction.

Capsular contracture is the most common adverse event in radiotherapy settings, and preventive measures are often unreliable [6]. Moreover, revisional surgeries usually fail to prevent recurrence. A prospective study by Spear et al. reported an incidence of capsular contracture of up to 15.9% in non-irradiated breasts [20]. However, in cases of adjuvant radiotherapy, the incidence significantly increases, ranging from 15% to 100% [5].

In the last decade, the use of acellular dermal matrices (ADMs) has been introduced in implant-based reconstruction to provide soft tissue coverage at the lower pole of the breast. This technique was first described by Karl H. Breuing in 2005, where he used AlloDerm (LifeCell Corp., Branchburg, NJ, USA) to cover the inferolateral pole of the implant in immediate reconstructions [10]. Over the years, various types of ADMs, sourced from both human and animal tissues, have been used in breast reconstruction, with NATIVE^®^ ADM recently showing favorable outcomes [21,22,23,24,25]. AlloDerm is a human-derived ADM, which makes it an allograft, while NATIVE is a porcine-derived ADM which makes it a xenograft. Both products are decellularized, thus making them biocompatible and able to integrate in host tissue, however, NATIVE is, in our experience, thicker, stronger and affords more support compared to Alloderm.

Several clinical studies have examined the use of ADMs to reduce the incidence of capsular contracture in immediate reconstructions, and a recent meta-analysis confirmed their effectiveness and reproducibility [26]. This benefit is likely due to the placement of ADM around the implant, which forms an antigen-free interface between the implant and host tissue. This interface may reduce the host immune response and capsule formation, while simultaneously providing support at the inferior pole [27,28].

Recent histological analyses have offered insights into the biological and immunological mechanisms by which ADMs reduce capsular formation, particularly by decreasing the proliferation of myofibroblasts and reducing tissue inflammation. This, in turn, minimizes excessive neovascularization and fibroblast migration into the capsular tissue, which can lead to thickening and fibrosis [15].

Because of this potential, ADMs have been used in the context of radiotherapy, and preliminary findings on their role in preventing radiation-related complications have been incorporated into the guidelines of the Association of Breast Surgery and the British Association of Plastic, Reconstructive, and Aesthetic Surgeons [29]. These guidelines recognize the negative impact of radiotherapy on implant-based breast reconstructions, but also suggest that ADMs may reduce the severity of capsular contracture.

Building on promising results with bovine ADMs [30], our group decided to implement porcine NATIVE^®^ ADM in irradiated breasts. Previous experience with bovine ADM was positive despite it being slightly thicker and less pliable than porcine ADM. With porcine ADM we observed a slightly lower complication rate. Especially major complications leading to reconstructive failure in our previous study occurred in 13.9% of patients against the 6.7% of the current study. Similarly, the infection rate was slightly higher in our previous study using bovine ADM (3.4% vs. 0%). NATIVE ADM was applied both to patients undergoing immediate reconstruction with an expander followed by post-mastectomy irradiation, and to those receiving direct-to-implant reconstructions who developed severe capsular contracture (Baker grade III-IV) after irradiation.

We propose this approach for selected patients where the flaps appear thick and well-vascularized, and in cases where patients prefer less invasive procedures without an absolute indication for autologous reconstruction, which remains the gold standard for irradiated breasts at our center.

In analyzing our data, we observed a 6.6% rate of major complications requiring implant removal. Notably, a systematic review by Mericli et al. on implant-based breast reconstruction in irradiated settings reports reconstructive failure rates ranging from 4.8% to 40% [31]. Our outcomes with ADM-assisted reconstructions fall at the lower end of this spectrum.

In our series, we analyzed patients with irradiated implants suffering from severe capsular contracture and those with irradiated expanders, noting significant differences. All four major complications and reconstructive failures occurred in Group A, consisting of patients who underwent expander reconstruction followed by radiotherapy. This increased risk profile is confirmed by the higher overall complication rate of 38.4% in Group A compared to 23.5% in Group B, where aesthetic outcomes were also significantly superior.

Our data suggest a safer profile for ADM use in implant substitution compared to expander substitution. This may be attributed to the greater need for pocket remodeling during the expander-to-implant exchange, which can lead to tissue devascularization, or the reduced blood perfusion and flap thickness in irradiated expanded tissues, which may hinder ADM integration.

In cases of capsular contracture, capsulotomy and capsulectomy are currently considered the gold standard treatment. However, recurrence rates remain high [32]. Data on the effectiveness of capsulectomy in preventing contracture are inconclusive. On the other hand, consistently low rates of capsular contracture recurrence have been observed with ADM use [33]. This is supported by Israeli’s work, which demonstrated that combining AlloDerm placement with capsulectomy provides an effective strategy to minimize recurrence in irradiated breasts [6].

Focusing on capsular contracture treatment, we observed a statistically significant reduction in recurrence among patients who underwent implant exchange and capsulectomy. In our highly selected patient population, porcine ADM combined with capsulectomy appeared to reduce the severity of capsular contracture, with promising postoperative outcomes.

The role of ADM in reducing capsular contracture in irradiated breasts has been previously described, notably by Sala et al., who reported no severe contracture in patients undergoing expander-to-implant replacement with porcine ADM, with all 88 patients improving to Baker grade I or II. Only two patients (5.8%) experienced implant loss in their cohort [34].

In this study, we identified two distinct patient populations and provided new information that contributes to the literature, enabling a better understanding of when ADM use offers the greatest benefits and the least complications.

This study has some limitations, primarily its retrospective nature and the relatively small patient cohort.

A retrospective design does not allow for randomization, making it impossible to draw definitive conclusions about differences in reconstructive outcomes and complications between the two groups, although the expander group did show a higher complication rate. However, our preliminary data suggest that final aesthetic outcomes were significantly better in the direct-to-implant group compared to the two-stage reconstructions. These findings should be considered in light of the prevailing view in many smaller breast reconstruction units that two-stage reconstruction remains the safer and better option when radiotherapy is planned.

The absence of a control group of patients who underwent reconstruction with implants without ADM prevents direct comparisons. This was unavoidable as all patients requiring breast reconstruction after radiotherapy at our center are offered either autologous reconstruction or ADM-assisted breast reconstruction. Nevertheless, our results are consistent with those reported in the literature, showing a reduction in unfavorable outcomes. Capsular contracture is a long-term complication. Although we followed all patients for at least one year, further follow-up at two and three years would be useful to ensure no long-term capsular contracture develops.

## 5. Conclusions

Regarding heterologous reconstruction in irradiated breasts, there is a notable lack of evidence in the literature concerning viable alternatives to autologous reconstruction. Consequently, the primary aim of this study is to demonstrate that acellular dermal matrix (ADM)-assisted breast reconstruction is a valid option, yielding satisfactory results for both patients requiring implant substitution and those undergoing expander-to-implant substitution despite prior irradiation.

Due to the retrospective nature of our study, definitive conclusions about the differences in outcomes between the two groups cannot be established. However, preliminary data suggest that in the context of adjuvant radiotherapy, direct-to-implant reconstruction may provide superior overall aesthetic outcomes, reduced complication rates, and a lower rate of failure compared to two-stage reconstruction (expander-to-implant).

Further research, ideally in the form of prospective and randomized studies, is required to confirm the long-term efficacy and safety of Native^®^-assisted breast reconstruction in the context of radiotherapy.

## Figures and Tables

**Table 1:** Patients demographics.

Clinical-Demographic Data	*N* (std)	%	*p*-Value
**Socio-demographic features**			
**Age (years, mean)** Group A Group B	52.6 (8.2) 51.8 (9)	-	**0.628**
**BMI (kg/m^2^)** Group A Group B	25.6 (1.9) 24.4 (2.5)	-	-
**Mean follow-up (months)**	20.2 (3.4)	-	-
**Smoking status** Active Smokers Group A Group B	3/26 0/34	11.5 0	**0.08**
**Diabetes**			
Group A Group B	0/26 0/34	0 0	**-**
**Type of mastectomy**			
**Nipple-sparing mastectomy** Group A Group B	11/26 13/34	42 38	**0.795**
**Skin-sparing mastectomy** Group A Group B	15/26 21/34	57 61	**0.795**
**Lymph nodes**			
**SLNB** Group A Group B	13/26 16/34	50 47	**1.00**
**Axillary Dissection** Group A Group B	13/26 18/34	50 53	**1.00**
**Chemotherapy**	84/84	100	
**Adjuvant** Group A Group B	23/26 32/34	88.5 94.2	**0.644**
**Neoadjuvant** Group A Group B	3/26 2/34	11.5 5.8	**0.644**
**Tumour histology (Group A 1 mixed ductal/lobular, Group B 2 undifferentiated**			
**Lobular** Group A Group B	9/26 14/34	34 41	**0.789**
**Ductal** Group A Group B	16/26 18/34	61 52	**0.602**
**Preop Capsular contracture on irradiated breasts**			
**Group A (Expander)** **Group B (Implant)** Grade III Grade IV	- 26/34 8/34	- 76.5 32.5	-

**Table 2:** Prior surgical history and surgical details of groups A and B.

Reconstructive Procedures (Unilateral in All pts)	*N* pts	%	*p*-Value
**Group A:** TE—Implant substitution (previous mastectomy and two-stage reconstruction) **Group B:** Implant substitution (previous mastectomy and IBR)	26 34	43.3 56.7	**0.201**
**Implant type**			
Round smooth Group A Group B	1/26 1/34	3.8 3	**1.00**
Anatomic textured Group A Group B	25/26 33/34	96.2 97	**1.00**
**Implant size**	**Mean (std)**	**Range**	
**Group A** **Group B**	445 cc (122) 448 cc (128)	235–690 270–690	**0.923**

**Table 3:** Complications and outcomes in pre-irradiated breasts and Native^®^ ADM assisted reconstructions.

Complications	N tot (60) (%)	Group A (26) (%)	Group B (34) (%)	*p*-Value
**Total complications**	15/60 (25)	8/26 (30.7)	7/34 (20.5)	**0.261**
**Major complications**	4/60 (6.7)	4/26 (15.4)	0/34 (0)	**0.03**
Implant exposure	2/60 (3.3)	2/26 (7.7)	0/34 (0)	**0.184**
Full thickness skin necrosis	1/60 (1.7)	1/26 (3.8)	0/34 (0)	**0.433**
Severe capsular contracture	1/60 (1.7)	1/26 (3.8)	0/34 (0)	**0.433**
**Minor Complications**	11/60 (18.3)	4/26 (15,)	7/34 (20.5)	**1.00**
Seroma	3/60 (5)	1/26 (3.8)	2/34 (5.9)	**1.00**
Superficial Skin necrosis	4/60 (6.7)	2/26 (7.7)	2/34 (5.9)	**1.00**
Hematoma	1/60 (1.7)	1/26 (3.8)	0/34 (0)	**0.433**
PMPS	2/60 (3.3)	0/26 (0)	2/34 (5.9)	**0.501**
Implant Rotation	1/60 (1.7)	0/26 (0)	1/34 (2.9)	**1.00**
**Reconstructive Failure**	4/60 (6.7)	4/26 (15.4)	0/34 (0)	**0.03**
**Capsular contracture after surgery**				
Grade I	6/60 (10)	4/26 (15.4)	2/34 (5.9)	**0.388**
Grade II	46/60 (76.7)	17/26 (65.4)	29/34 (85.3)	**0.122**
Grade III	3/60 (5)	2/26 (7.7)	1/34 (2.9)	**0.574**
Grade IV	1/60 (1.7)	1/26 (3.8)	0/34 (0)	**0.433**
Not Determined	4/60 (6.7)	2/26 (7.7)	2/34 (5.9)	**1.00**

**Table 4:** Level of satisfaction among surgeons for Group A and B.

Surgeons’ Satisfaction	Total *n* (%)	Group A *n* (%)	Group B *n* (%)	*p* Value
**Highly satisfied**	46/60 (76.6)	16/26 (61.5)	30/34 (88.2)	**0.03**
**Moderately satisfied**	8/60 (13.3)	4/26 (15.4)	4/34 (11.8)	**0.717**
**Unsatisfied**	2/60 (3.3)	2/26 (7.7)	0/34 (0)	**0.184**
**Not assessable**	4/60 (6.7)	4/26 (15.4)	0/34 (0)	**0.03**
**Tot n breasts**	60	26	34	

## Data Availability

The data that support the findings of this study are available on request from the corresponding author.
